# Ultrasound-Guided Transmuscular Quadratus Lumborum Block Reduces Postoperative Pain Intensity in Patients Undergoing Total Hip Arthroplasty: A Randomized, Double-Blind, Placebo-Controlled Trial

**DOI:** 10.1155/2020/1035182

**Published:** 2020-03-07

**Authors:** Jian He, Lei Zhang, Wan You He, Dong Lin Li, Xue Qin Zheng, Qi Xia Liu, Han Bin Wang

**Affiliations:** Department of Anesthesiology, The First People's Hospital of Foshan, Foshan City, China

## Abstract

**Methods:**

Eighty-eight patients undergoing THA were randomized to receive 0.33% ropivacaine (Group QLB, *n* = 44) or saline (Group Con, *n* = 44) for QL3 block. Spinal anesthesia was then performed. Pain intensity was assessed using the visual analog scale (0: no pain to 10: worst possible pain). The primary outcome was pain scores recorded at rest at 3, 6, 12, 24, 36, and 48 h and on standing and walking at 24, 36, and 48 h postoperatively. Secondary outcomes were analgesic consumption, side effects, the 10-meter walking speed on day 6, and patient satisfaction after surgery.

**Results:**

Postoperative pain intensity was significantly lower in Group QLB compared to Group Con at rest after 3, 6, 12, 24, 36, and 48 h (*p* < 0.001) and during mobilization after 24, 36, and 48 h (*p* < 0.001). Morphine use was significantly lower in Group QLB compared to Group Con during 0–24 h (16.0 ± 7.1 vs. 34.1 ± 7.1 mg, *p* < 0.001) and during 24–48 h (13.0 ± 4.0 vs. 17.4 ± 4.6 mg, *p* < 0.001) postoperatively. The 10-meter walking speed was higher in Group QLB compared to Group Con, both at comfortable (0.79 ± 0.13 vs. 0.70 ± 0.14 m/s, *p*=0.012) and at maximum speeds (1.18 ± 0.26 vs. 1.06 ± 0.22 m/s, *p* < 0.001). Incidences of nausea (7.3% vs. 31%, *p*=0.006), vomiting (7.3% vs. 26.2%, *p* = 0.022), and urinary retention (9.8% vs. 28.6%, *p*=0.030) were lower in Group QLB than in Group Con.

**Conclusions:**

Ultrasound-guided QL3 block is an effective pain management technique after THA.

## 1. Introduction

Many patients with total hip arthroplasty (THA) experience moderate to severe acute pain in the early postoperative period [1]. Effective postoperative pain management can ensure early participation in rehabilitation and increase patient satisfaction [1]. Otherwise, patients may suffer chronic pain and disability, with a poor quality of life [2, 3].

There are many methods to manage postoperative pain following THA, including the use of opioids and nonsteroidal anti-inflammatory drugs, local infiltration analgesia, patient-controlled analgesia, and peripheral nerve blocks (PNBs) [4].The use of opioids, either intravenously or epidurally, can cause itching, urinary retention, respiratory depression, and other side effects. In recent years, multimodal pain management strategy by targeting multiple different pain pathway mechanisms has been recommended to reduce opioid use and opioid-related adverse effects [5, 6]. With the developments in ultrasound techniques, PNBs have become popular for postoperative pain control. Peripheral nerve blocks, including fascia iliaca block, femoral nerve block (FNB), lateral femoral cutaneous nerve block, and lumbar plexus block have been used for postoperative pain management after THA [4, 7, 8]. However, the best postoperative pain management technique after THA remains uncertain. Sensory innervation of the hip joint is complex, and mainly includes the femoral, obturator, and lateral femoral cutaneous nerves anteriorly and the sciatic nerve posteriorly. Single nerve blocks may have poor analgesic effects in THA, and therefore, the lumbar plexus block may be an ideal PNB for postoperative pain control following THA. However, the lumbar plexus block requires operator expertise, takes a longer time to perform, and has a greater risk of complications [9]. Thus, better analgesic strategies are required for patients after THA and should be investigated.

The quadratus lumborum (QL) block was initially reported for postoperative pain control by Blanco in 2007 [10]. There are five approaches including type 1 (QL1), type 2 (QL2), transmuscular approach (QL3), intramuscular approach, and paramedian sagittal oblique approach according to injection location. Previous studies have shown that QL1 and QL2 blocks may generate analgesia from T7 to L1 [11, 12],and the QL3 block may cause caudal spread to the L2–L3 dermatomes [13]. Thus, the QL3 approach may be more suitable for hip surgery than the other approaches. Several studies have demonstrated that the QL block has a long-lasting analgesic effect on patients after surgery [11, 12, 14, 15]. Furthermore, two case reports showed that the QL block was a good method for postoperative pain relief after THA [16, 17]. However, case reports, by themselves, do not have enough data to draw conclusions. We designed this prospective, randomized, double-blind, placebo-controlled trial to investigate whether the QL3 block can be used for analgesia after THA.

The aim of this placebo-controlled trial was to investigate the effects of the QL3 block on pain intensity, opioid requirement, and mobilization in patients undergoing THA. We hypothesized that the QL3 block would reduce pain intensity and opioid requirements in patients following THA.

## 2. Methods

### 2.1. Patients and Methods

The study was approved by the China Ethics Committee of Registering Clinical Trials (ChiECRCT-20170094) before the first patient recruitment. It was registered in the China Clinical Trial Registry on December 18, 2018 (Registration number ChiCTR1800014295). All patients and one of the family members gave verbal and written informed consent before inclusion in the study. This was a single-center, prospective, randomized, double-blind, placebo-controlled, parallel-group study carried out in the First People's Hospital of Foshan in China from January 25, 2018, to August 1, 2018 (last patient follow-up). No changes were made to the study protocol after the start of the study. Patients with ASA status I–III, aged 18–75 years, with a diagnosis of osteoarthritis or osteonecrosis and undergoing elective unilateral THA were included in the study. Exclusion criteria were as follows: weight less than 30 kg or more than 100 kg; allergy to local anesthetics; known history of intolerance to drugs used in the study; psychiatric illnesses; local skin infections at the puncture points; peripheral neuropathy; sensory disorders in the leg requiring surgery; coagulation abnormalities; opioid abuse; chronic pain; severe liver, heart, and kidney impairment; and inability to understand and use patient-controlled pump for analgesia. All patients stopped taking nonsteroidal anti-inflammatory drugs or acetylsalicylic acid prior to surgery. Patients were given instructions about the visual analog scale (VAS) for pain assessment, with scores ranging from 0 to 10 (0 = no pain, 10 = worst imaginable pain) and the use of the patient-controlled analgesia (PCA) morphine device prior to anesthesia.

### 2.2. Randomization and Blinding

Randomization was performed by concealed allocation using a random number table to generate a randomization list; these were inserted into sequentially numbered, opaque, sealed envelopes. A total of 88 consecutive numbered envelopes (44/group) were thus made by staff with no further involvement in this study. The randomization list was kept in a locked iron sheet cabinet and was not accessible to the staff involved in the study. Subjects included in the study were assigned to receive treatment based on the randomization list. The ropivacaine and saline solutions were prepared in a syringe and labeled with the patient's id number by a nurse according to the randomization list; the two solutions were identical in appearance. This process was verified by a second nurse. This personnel was not further involved in evaluating or treating patients in this trial. The QL3 block was completed in the preanesthesia room. The surgeon, anesthesiologist, and other operating room staff did not know which solution was being used. Data collection was performed in a double-blinded manner, such that neither the patients nor the health-care personnel was aware of the medication assigned.

### 2.3. Anesthesia and Analgesia

**Figure 1:**
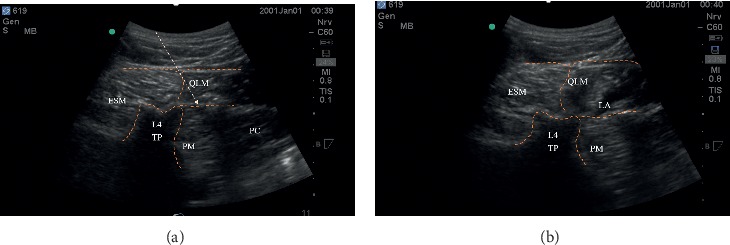
(a) The QL3 block. (a) The ultrasound image before injection. Arrow indicates needle trajectory and injection point between QLM (quadratus lumborum muscle) and PM (psoas major muscles). ESM: erector spinae muscle; L4 TP: L4 transverse process; PC: peritoneal cavity. (b) The QL3 block. Figure 1(b) shows the ultrasound image after injection. Arrow indicates needle trajectory and injection point between QLM (quadratus lumborum muscle) and PM (psoas major muscles). ESM: erector spinae muscle; L4 TP: L4 transverse process; PC: peritoneal cavity. LA: local anesthetics.

The quadratus lumborum (QL) block with the type 3 approach was performed by the same anesthesiologist as a single injection before surgery in the preanesthesia room. The anesthesiologist had performed more than 50 QL3 blocks before participating in this study. This experienced anesthesiologist (J. H.) was blinded to the injectate. According to the previously described QL3 block technique [18], with the patient in the lateral decubitus position, a curvilinear transducer (2–5 MHz) was placed transversely in the midaxillary line above the iliac crest. In this position, the quadratus lumborum is anterolateral to the apex of the L3 and L4 transverse processes with the psoas major muscle anteriorly and the erector spinae muscles posteriorly. After the skin was cleaned and prepared, a 120 mm, 22-gauge Tuohy needle was inserted in-plane under the ultrasound beam from the posterior to the anterior direction through the quadratus lumborum muscle until the ventral fascia of the muscle was penetrated (Figures 1(a) and 1(b)). The right needle tip position was confirmed by injection of 1-2 ml of normal saline solution that spread between the quadratus lumborum and psoas major muscles. Thereafter, according to group randomization, 0.33% ropivacaine or saline was injected, with the volume of injection depending on the patient's body weight.

In Group QLB, 30 ml of 0.33% ropivacaine (containing 30 micrograms of dexmedetomidine and 5 mg of dexamethasone) was administered in patients with a body weight of >75 kg, 25 ml 0.33% ropivacaine (containing 25 micrograms of dexmedetomidine and 5 mg of dexamethasone) in patients with a body weight of 50–75 kg, or 20 ml 0.33% ropivacaine (containing 20 micrograms of dexmedetomidine and 5 mg of dexamethasone) in patients with a body weight of 30–50 kg. The principle of the volume of saline injection in the control group (Group Con) was the same as that in the QLB group.

Spinal anesthesia was then performed with 0.5% ropivacaine, 1.8–2.5 ml, depending on patient characteristics. Participants were excluded from the trial in case of failure of spinal anesthesia. Perioperative anesthesia management was carried out according to our departmental guidelines.

### 2.4. Surgical Technique

All surgical procedures were performed in a standardized manner through the posterolateral approach. A longitudinal skin incision was made over the greater trochanter. The gluteal fascia and the iliotibial band were dissected, and the insertion of the gluteus medius was divided down to the bone. The capsule was then exposed and incised, and thereafter the surgical procedure was standard. Bone cement was not used in any of the subjects included in the study. Surgeons were blinded to the patient grouping and performed rehabilitation training after the operation. The same postoperative care and physical therapy regimen were provided to all patients.

### 2.5. Preoperative and Postoperative Pain Management

All subjects received the same perioperative pain management. All patients received 1000 mg intravenous (IV) acetaminophen 1 hour prior to surgery. In the orthopedic ward, patients were administered 40 mg parecoxib IV, every 12 h for 2 days, and acetaminophen 500 mg orally every 6 h after the operation until the patient was discharged from the hospital. Additionally, a PCA device was provided as rescue medication with IV morphine injection 1 mg when required with a 10 min lock-out time. After 48 hours, the PCA device was removed, and all patients then received 10 mg sustained-release oxycodone p.o. twice a day until discharge. This is the standard postoperative analgesia protocol used in our hospital.

### 2.6. Recordings and Measurements

All patients were instructed to assess the intensity of their pain using the VAS, with scores ranging from 0 (no pain) to 10 (the worst possible pain). The primary outcome was pain scores recorded at rest at 3, 6, 12, 24, 36, and 48 h after surgery, and on standing and walking at 24, 36, and 48 h postoperatively by two specially trained assistants (one nurse and one resident doctor). Secondary outcomes included the following:

1. Analgesic consumption: total morphine consumption by each patient during 0–24 h and 24–48 h after surgery.
2. Bradycardia, hypotension, and respiratory depression (respiratory rate < 8 breaths/min) during operation.
3. Side effects: postoperative nausea, vomiting, pruritus, respiratory depression (respiratory rate < 8/min), and urinary retention from 0 to 48 h after surgery.
4. The 10-meter walk test: this test was used to assess the patient's motor functions on day 2 and day 6 after the operation. The patient walked 10 meters in a straight line at the most comfortable pace, and the time required was recorded. Fifteen minutes later, the patient walked 10 meters in a straight line as quickly as possible, and the time required was recorded. The walking process was carried out twice, and the walk that was completed quicker was included for the analysis [19]. Gait speeds were expressed in meters per second.
5. Patient satisfaction: this was assessed on day 6 after surgery using the following scale: 1 = terrible, 2 = poor, 3 = satisfactory, 4 = good, and 5 = excellent.

### 2.7. Statistical Analysis

Our pilot study showed that the mean pain VAS score at 12 h was 4 (standard deviation [SD] 3). Using *α* = 80% and *β* = 0.05, a 2-tailed analysis showed that we needed 36 patients/group for a reduction in pain intensity by 50%. We planned to recruit a total of 88 patients to compensate for 20% dropouts. Continuous variables were summarized as mean and SD or as median and interquartile range. Categorical variables were described using frequencies or proportions. To test if statistically significant differences existed between the two randomized groups, an independent *t*-test for nonnormally distributed outcome variables was used for continuous outcomes. Chi-square test or Fischer's exact test was used to compare the study groups for categorical data, such as side effects and complications. A repeated-measures analysis of variance (ANOVA) was used to estimate the difference in VAS scores between the two groups at each time point. *p* values < 0.05 were considered to be statistically significant. All statistical analyses were performed using SPSS version 16. After completion of the study, the data were typed into a spreadsheet by two researchers. A randomization list assigning subjects to either group “a” or “b” was created without revealing the identity of the groups. The statistical analysis was completed, and conclusions were drawn before it was revealed as to which group received ropivacaine and which received a placebo.

## 3. Results

**Figure 2:**
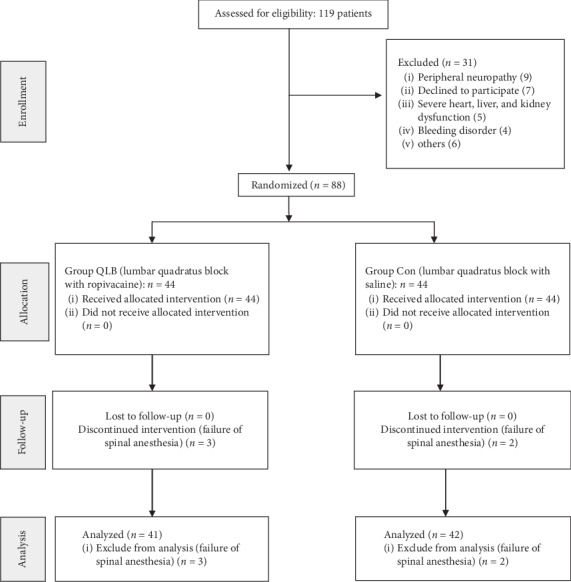
Consolidated standards of reporting trials flow diagram.

Our intention was to recruit 88 patients although power calculations suggested that 72 patients would be adequate to analyze the primary outcome measure. The CONSORT diagram for patient recruitment is shown in Figure 2. Five patients were excluded after randomization because of the failure of spinal anesthesia. There were no statistically significant differences in demographic data and operation characteristics between the two groups (Table 1).

Values are shown as mean (SD) or number; Group QLB = quadratus lumborum block; Group Con = control group; ASA physical status: I = normal healthy patient, II = patient with mild systemic disease, III = patient with severe systemic disease, *n* = number of patients.

With regard to pain management, a statistically significant decrease in pain intensity was observed in the QLB group. Regarding the primary outcome, patients in Group QLB had significantly lower VAS scores at rest at 3, 6, 12, 24, 36, and 48 h after surgery compared to Group Con (*p* < 0.001). Patients in Group QLB also had lower pain scores during mobilization at 24, 36, and 48 h compared to Group Con (*p* < 0.001). Pain intensity in patients at 3, 6, 12, 24, 36, and 48 hours after the operation during rest and mobilization are shown in Table 2.

**Figure 3:**
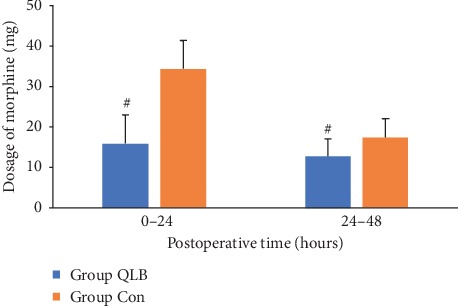
The dosage of morphine consumption. Data are presented as mean (SD), Group QLB = quadratus lumbar block, Group Con = control group, ^#^*p* < 0.001.

The dosage of opioid demand can also indicate pain control after the operation. On the first day after surgery, morphine consumption was significantly decreased in the QLB group compared to the control group (mean, 16.0 vs. 34.0 mg, *p* < 0.001). There was also a significantly lower morphine consumption in the QLB group than in the control group on day 2 after surgery (mean, 13.0 vs. 17.4 mg, *p* < 0.001) (Table 3, Figure 3).

Regarding the 10-meter walk test used to measure the time taken for the patient to walk in a straight line for 10 meters at comfortable and maximum speeds, only 13 patients completed the test on day 2 after the operation. Therefore, there were no enough data for statistical analysis on day 2 after the operation. The 10-meter walking speed was significantly higher in Group QLB than in the control group, both at comfortable (0.79 ± 0.13 vs. 0.70 ± 0.14 m/s, *p*=0.012) and at maximum speeds (1.18 ± 0.26 vs. 1.06 ± 0.22 m/s, *p* < 0.001) on day 6 after the operation (Table 4).

Bradycardia, respiratory depression, and hypotension were not observed in the two groups intra-/postoperatively. The incidences of nausea (7.3% vs. 31%, *p*=0.006), vomiting (7.3% vs. 26.2%, *p*=0.022), and urinary retention (9.8% vs. 28.6, *p*=0.030) were significantly decreased in Group QLB compared to Group Con (Table 5). No significant differences were observed in the incidence of pruritus after the operation in the two groups.

Patient satisfaction was significantly higher in Group QLB than in Group Con (3.7 ± 0.8 vs. 2.8 ± 0.9, *p* < 0.001) on day 6 after surgery.

## 4. Discussion

The results of this study show that quadratus lumborum block type 3 (QL3) using a solution that combined ropivacaine, dexamethasone, and dexmedetomidine can significantly reduce the intensity of postoperative pain, both at rest and on movement in the first 48 h after THA. Additionally, better physical performance, as evaluated by the 10 meters walking speed, was noticed in patients receiving QL3 block compared to patients in the control group as well. The incidence of urinary retention, nausea, and vomiting after THA was lower in the QLB group compared to the control group.

Surgical incision is the main source of postoperative pain. Hip innervation is complex, and the nerves involved during incision of the THA mainly include the lateral femoral cutaneous nerve, femoral nerve, obturator nerve, and sciatic nerve [20]. Many studies have shown that blockade of any of these nerves can reduce pain scores and opioid use in patients undergoing THA. However, blocking more nerves innervating the hip joint might provide better postoperative pain relief. Moreover, the latest study on the anatomy of the hip nerves shows that the femoral nerve may branch out at a very high position, and it dominates the hip joint [21]. In addition, in nearly 50% of patients, the accessory obturator nerve innervated the hip at the position where the obturator nerve had just emerged [21]. Therefore, the conventional method of blocking the femoral and obturator nerves in the inguinal region may not completely block the femoral nerve branches and the accessory obturator nerve that dominates the hip joint. Lumbar plexus block may be an ideal postoperative pain management strategy after THA; however, it requires more expertise and is riskier than single nerve blocks.

Blanco first reported that the QL block was an extremely effective method of postoperative pain control in 2007 [10]. There are several approach methods: type 1 (QL1), type 2 (QL2), transmuscular approach (QL3), intramuscular approach, and paramedian sagittal oblique approach [22]. The spread of local anesthetic varies with each approach. Previous case reports have shown that both QL1 and QL3 can provide good pain relief in patients following THA [16, 17, 23]. Moreover, a prospective controlled study by Parras and Blanco indicated that QL1 provided better pain relief than femoral nerve block in patients with femoral neck fractures [24]. However, the most suitable approach of QL block for analgesia undergoing THA is yet to be determined. A cadaver study showed that after QL3 block, the injectate spread consistently to L1, L2, and L3 nerve roots, which are important components of the lumbar plexus [13]. Therefore, the QL3 block may provide analgesia covering the dermatomes extending caudally to L2 or L3 and is an effective pain management strategy in hip surgeries. However, the mechanism of the QL3 block for postoperative analgesia is still controversial. A cadaver study by Dam et al. showed that after QL3 block, the injectate could spread into the thoracic paravertebral space and the intercostal spaces to surround the somatic nerves and the thoracic sympathetic trunk through the thoracolumbar fascia [25]. However, other cadaver studies have shown that the injectate could not spread into the thoracic paravertebral space after the QL3 block [13, 26]. In addition, all these studies have demonstrated that the QL3 blockade can spread into the region surrounding the upper branches of the lumbar plexus. Although controversy exists on whether the local anesthetic spreads into the thoracic paravertebral space after the QL3 blockade, it is clear that the QL3 approach can block part of the lumbar plexus, and thus plays a role in postoperative analgesia. Moreover, the QL3 block is less invasive, safer, and easier to perform than a lumbar plexus block, which requires injection within the psoas muscle adjacent to the roots of the lumbar plexus [27].

Generally speaking, the duration of action of a single nerve block is no longer than 24 hours. However, in our study, the VAS scores and the dosage of morphine required in the QLB group were lower than those in the control group on day 2 after the operation. There may be several reasons to account for these results. First, the QL3 block acts on the muscular fascia, and its duration of action is possibly longer than that of other nerve blocks. Since nerves are often accompanied by blood vessels, local anesthetic injected along the nerve is absorbed more quickly than local anesthetic injected in the muscular fascia. The peak arterial ropivacaine levels at comparable times were significantly lower with QL block than with lateral TAP block, and the median duration of analgesia after QL block was longer than that after the TAP block [28]. This suggests that local anesthetic may be absorbed more slowly by QL injection than by TAP injection. Secondly, dexmedetomidine and dexamethasone may prolong the duration of action of local anesthetics. La Colla et al. reported a case wherein complete sensory block was present for more than 24 hours after QL block with ropivacaine, dexmedetomidine, and dexamethasone [16]; these findings are consistent with our study. Finally, QL block can effectively inhibit pain in 24 hours postoperatively and reduce postoperative pain sensitization, while patients in the control group had higher VAS score at 24–48 hours after surgery. Blanco found that the duration of the QL block was about 48 hours, which is similar to what we found in this study [12].

The 10-meter walk test was used to evaluate the patients' physical performance. The speed in the test in the QLB group was higher than that in the control group, both at comfortable and at maximum speeds. Good postoperative analgesia and lower incidence of opioid-related side effects made patients more willing to perform rehabilitation exercises. Moreover, patients were more likely to walk faster during the test due to lower pain levels. These reasons likely accounted for the better physical performance in the QLB patient group than in the control group.

The incidences of nausea, vomiting, and urinary retention in patients who received QLB were significantly decreased in this study. These opioid-related side effects may affect the patient's ability to perform functional exercise and rehabilitation, which may also decrease patients' satisfaction as well. Effective analgesia and fewer opioid-related side effects played a pivotal role in improving satisfaction in patients receiving the QL3 block. This study did not find QL3 block related hypotension or respiratory depression.

There were several limitations to our study that should be considered. First, no sensory testing was performed in this trial. Sensory testing is a routine practice at our institution, but we decided not to perform this in order to avoid unblinding of the study. Secondly, we did not evaluate the strength of the quadriceps femoris muscle. Weakening of quadriceps femoris muscle strength is an important factor contributing to falls after hip and knee arthroplasties [29]. Some cases of QL1 block with lower limb weakness have been reported [30]. The mechanisms of QL3 and QL1 blocks are similar to some degree. Thus, studies with a larger sample should be carried out to evaluate the quadriceps femoris muscle strength after the QL3 block.

## 5. Conclusions

In conclusion, we have demonstrated that ultrasound-guided QL3 block is an effective pain management technique after THA. Further trials are required to evaluate the quadriceps femoris muscle strength after the QL3 block.

## Figures and Tables

**Table 1:** Demographic data and duration of surgery.

Patient characteristics	Group Con	Group QLB	*p* value
Female/male	31/11	28/13	0.579
Age, *y*	67 (8)	66 (7)	0.382
Weight, kg	57 (7.5)	57 (8.2)	0.898
Height, cm	161 (6.6)	163 (6.4)	0.094
ASA, I/II/III (*n*)	3/29/10	5/28/8	0.451
Operation time, minutes	100 (7)	98 (8)	0.269

**Table 2:** Pain scores at 3, 6, 12, 24, 36, and 48 hours after surgery.

	Group QLB (*n* = 41)	Group Con (*n* = 42)	*p* value
VAS scores at rest (0–10)			
3 h postoperative	0.85 ± 0.53	1.04 ± 0.86	0.001
6 h	1.32 ± 0.72	2.74 ± 1.04	<0.001
12 h	2.51 ± 0.95	3.81 ± 0.77	<0.001
24 h	2.39 ± 0.83	3.38 ± 0.96	<0.001
36 h	1.83 ± 0.59	2.50 ± 0.71	<0.001
48 h	1.56 ± 0.50	2.38 ± 0.73	<0.001

VAS scores at mobilization (0–10)			
24 h postoperative	3.02 ± 1.06	6.10 ± 1.48	<0.001
36 h	3.07 ± 0.84	5.45 ± 1.13	<0.001
48 h	2.12 ± 0.64	4.33 ± 0.93	<0.001

Values are shown as mean ± SD; Group QLB = quadratus lumbar block; Group Con = control group; VAS = visual analog score.

**Table 3:** Postoperative morphine consumption.

	Group QLB (*n* = 41)	Group Con (*n* = 42)	*p* value
Morphine consumption (mg)			
0–24 h after surgery	16.0 (7.1)	34.1 (7.1)	<0.001
24–48 h after surgery	13.0 (4.0)	17.4 (4.6)	<0.001

Values are shown as mean (SD); Group QLB = quadratus lumbar block; Group Con = control group.

**Table 4:** The 10-meter walk speed at comfortable and maximum speeds on day 6 after the operation.

	Group QLB (*n* = 41)	Group Con (*n* = 42)	*p* value
10 m walking speed, at comfortable pace (m/s)	0.79 ± 0.13	0.70 ± 0.14	0.012
10 m walking speed, at maximum pace (m/s)	1.18 ± 0.26	1.06 ± 0.22	0.026

Values are shown as mean ± SD; Group QLB = quadratus lumborum block; Group Con = control group.

**Table 5:** Side effects after surgery.

	Group QLB (*n* = 41)	Group Con (*n* = 42)	*p* value
Nausea, *n* (%)	3 (7.3)	13 (31)	0.006
Vomiting, *n* (%)	3 (7.3)	11 (26.2)	0.022
Pruritus, *n* (%)	2 (4.9)	6 (14.3)	NS
Urinary retention	4 (9.8)	12 (28.6)	0.030
Respiratory depression	0 (0)	0 (0)	NS

*n* = number of patients. Group QLB = quadratus lumborum block; Group Con = control group; NS = not significant.

## Data Availability

The data used to support the findings of this study are included within the article.

## References

[1] Parvataneni H. K., Shah V. P., Howard H., Cole N., Ranawat A. S., Ranawat C. S. (2007). Controlling pain after total hip and knee arthroplasty using a multimodal protocol with local periarticular injections. *The Journal of Arthroplasty*.

[2] Dalury D. F., Lieberman J. R., MacDonald S. J. (2011). Current and innovative pain management techniques in total knee arthroplasty. *The Journal of Bone & Joint Surgery*.

[3] Gerbershagen H. J., Pogatzki-Zahn E., Aduckathil S. (2014). Procedure-specific risk factor analysis for the development of severe postoperative pain. *Anesthesiology*.

[4] Højer Karlsen A. P., Geisler A., Petersen P. L., Mathiesen O., Dahl J. B. (2015). Postoperative pain treatment after total hip arthroplasty: a systematic review. *Pain*.

[5] Hebl J., Dilger J., Byer D. (2008). A pre-emptive multimodal pathway featuring peripheral nerve block improves perioperative outcomes after major orthopedic surgery. *Regional Anesthesia and Pain Medicine*.

[6] Tayrose G., Newman D., Slover J., Jaffe F., Hunter T., Bosco J. (2013). Rapid mobilization decreases length-of-stay in joint replacement patients. *Bulletin of the Hospital for Joint Disease (2013)*.

[7] Singelyn F., Ferrant T., Malisse M., Joris D. (2005). Effects of intravenous patient-controlled analgesia with morphine, continuous epidural analgesia, and continuous femoral nerve sheath block on rehabilitation after unilateral total-hip arthroplasty. *Regional Anesthesia and Pain Medicine*.

[8] Wiesmann T., Steinfeldt T., Wagner G., Wulf H., Schmitt J., Zoremba M. (2014). Supplemental single shot femoral nerve block for total hip arthroplasty: impact on early postoperative care, pain management and lung function. *Minerva Anestesiologica*.

[9] Capdevila X., Coimbra C., Choquet O. (2005). Approaches to the lumbar plexus. *Regional Anesthesia and Pain Medicine*.

[10] Blanco R. (2007). Tap block under ultrasound guidance: the description of a “no pops” technique. *Regional Anesthesia and Pain Medicine*.

[11] Murouchi T., Iwasaki S., Yamakage M. (2016). Quadratus lumborum block. *Analgesic Effects and Chronological Ropivacaine Concentrations after Laparoscopic Surgery*.

[12] Blanco R., Ansari T., Girgis E. (2015). Quadratus lumborum block for postoperative pain after caesarean section. *European Journal of Anaesthesiology*.

[13] Carline L., Mcleod G. A., Lamb C. (2016). A cadaver study comparing spread of dye and nerve involvement after three different quadratus lumborum blocks. *British Journal of Anaesthesia*.

[14] Kadam V. (2013). Ultrasound-guided quadratus lumborum block as a postoperative analgesic technique for laparotomy. *Journal of Anaesthesiology Clinical Pharmacology*.

[15] Visoiu M., Yakovleva N. (2013). Continuous postoperative analgesia via quadratus lumborum block—an alternative to transversus abdominis plane block. *Pediatric Anesthesia*.

[16] La Colla L., Uskova A., Ben-David B. (2017). Single-shot quadratus lumborum block for postoperative analgesia after minimally invasive hip arthroplasty. *Regional Anesthesia and Pain Medicine*.

[17] Ueshima H., Yoshiyama S., Otake H. (2016). The ultrasound-guided continuous transmuscular quadratus lumborum block is an effective analgesia for total hip arthroplasty. *Journal of Clinical Anesthesia*.

[18] Jadon A., Motka M., Kumar Pati A., Sinha N. (2016). Postoperative analgesia by transmuscular quadratus lumborum block catheters. *Journal of Anesthesia & Intensive Care Medicine*.

[19] Watson M. J. (2002). Refining the ten-metre walking test for use with neurologically impaired people. *Physiotherapy*.

[20] Amlong C. A., Schroeder K. M., Andrei A. C., Han S., Donnelly M. J. (2012). The analgesic efficacy of transversus abdominis plane blocks in ileostomy takedowns: a retrospective analysis. *Journal of Clinical Anesthesia*.

[21] Short A. J., Barnett J. J. G., Gofeld M. (2018). Anatomic study of innervation of the anterior hip capsule: implication for image-guided intervention. *Regional Anesthesia and Pain Medicine*.

[22] Hironobu U., Hiroshi O., Jui-An L. (2017). Ultrasound-guided quadratus lumborum block: an updated review of anatomy and techniques. *BioMed Research International*.

[23] La Colla L., Ben-David B., Merman R. (2017). Quadratus lumborum block as an alternative to lumbar plexus block for hip surgery. *A & A Case Reports*.

[24] Parras T., Blanco R. (2016). Randomised trial comparing the transversus abdominis plane block posterior approach or quadratus lumborum block type I with femoral block for postoperative analgesia in femoral neck fracture, both ultrasound-guided. *Revista Española de Anestesiología y Reanimación (English Edition)*.

[25] Dam M., Moriggl B., Hansen C. K., Hoermann R., Bendtsen T. F., Børglum J. (2017). The pathway of injectate spread with the transmuscular quadratus lumborum block. *Anesthesia & Analgesia*.

[26] Adhikary S. D., El-Boghdadly K., Nasralah Z., Sarwani N., Nixon A. M., Chin K. J. (2017). A radiologic and anatomic assessment of injectate spread following transmuscular quadratus lumborum block in cadavers. *Anaesthesia*.

[27] Mannion S., Barrett J., Kelly D., Murphy D. B., Shorten G. D. (2005). A description of the spread of injectate after psoas compartment block using magnetic resonance imaging. *Regional Anesthesia and Pain Medicine*.

[28] Blanco R., Ansari T., Riad W., Shetty N. (2016). Quadratus lumborum block versus transversus abdominis plane block for postoperative pain after cesarean delivery. *Regional Anesthesia and Pain Medicine*.

[29] Ilfeld B. M., Duke K. B., Donohue M. C. (2010). The association between lower extremity continuous peripheral nerve blocks and patient falls after knee and hip arthroplasty. *Anesthesia & Analgesia*.

[30] Wikner M. (2017). Unexpected motor weakness following quadratus lumborum block for gynaecological laparoscopy. *Anaesthesia*.

